# Rapid identification and antimicrobial susceptibility testing of pathogenic microorganisms from positive blood cultures

**DOI:** 10.1371/journal.pone.0358947

**Published:** 2026-09-23

**Authors:** Wenna Xu, Xiaomin Liu, Nansong Xu, Xiaohui Li, Fangqi Liu, Qianyan Li, Minqi Li, Zulu Ye, Xin Zheng

**Affiliations:** 1 Department of Clinical Laboratory, State Key Laboratory of Oncology in South China, Collaborative Innovation Center for Cancer Medicine, Guangdong Provincial Clinical Research Center for Cancer, Sun Yat-sen University Cancer Center, Guangzhou, China; 2 School of Medical Technology, Guangdong Medical University, Dongguan, China; 3 Yunkang School of Medicine and Health, Nanfang College Guangzhou, Guangzhou, China; 4 Department of Molecular Diagnostics, State Key Laboratory of Oncology in South China, Collaborative Innovation Center for Cancer Medicine, Guangdong Provincial Clinical Research Center for Cancer, Sun Yat-sen University Cancer Center, Guangzhou, China; Tribhuvan University, NEPAL

## Abstract

**Purpose:**

Bloodstream infections (BSIs) are severe illnesses associated with high mortality. Delays in obtaining microbial culture results often hinder timely targeted antimicrobial therapy. Our study aimed to evaluate the feasibility of rapid pathogen identification (ID) and antimicrobial susceptibility testing (AST) in BSIs.

**Methods:**

Positive blood culture (BC) broth was transferred into separation gel vacuum blood collection tubes for preprocessing, followed by pathogen identification via matrix-assisted laser desorption/ionization time-of-flight mass spectrometry (MALDI-TOF MS). Identification concordance rates (ICRs) were calculated against the conventional reference method. Bacterial membrane rapid AST (BMR-AST) and positive blood culture medium rapid AST (PBCMR-AST) were performed using cultured bacterial membrane and raw positive BC medium, respectively, with results compared to conventional method.

**Results:**

A total of 210 specimens were analysed for rapid ID. ICRs were 74.6% (88/118) for Gram-negative bacteria (GNB), 37.2% (29/78) for Gram-positive bacteria (GPB), and 14.3% (2/14) for fungi. BMR-AST yielded category agreement (CA) >96% for 58 GNB (96.8%, 1125/1162) and 24 Gram-positive cocci (GPC, 97.7%, 334/342), with essential agreement (EA) above 97% for both groups. PBCMR-AST achieved 94.1% CA (270/287) among 65 *Enterobacterales*; overall CA reached 94.7% (557/588) across 70 GNB and 28 GPC for additional antimicrobials.

**Conclusion:**

This rapid ID workflow performed well for GNB but suboptimally for GPB and fungi. Both rapid ASTs showed excellent concordance with the standard method for both GNB and GPC. These approaches can substantially shorten the turnaround time of positive BCs and hold promising clinical value for early BSI management.

## 1. Introduction

Among severe diseases, bloodstream infections (BSIs) are one of the most common causes of death [1]. A survey revealed that the case-fatality rate of nosocomial BSI in Europe ranges from 12% to 32%, with more than 29,000 deaths per year [2]. The latest analyses from the World Health Organization (WHO) revealed that sepsis affects 49 million individuals worldwide and is responsible for approximately 11 million potentially avoidable deaths [3]. The survival rate of patients with sepsis decreases by nearly 8% for each hour of delayed treatment [4]. Blood culture (BC) remains the gold standard for diagnosing clinical BSI, but results are often not available for 2–3 days, as positive BCs require subculture onto solid agar plates to obtain pure isolated colonies [5–7]. Early and appropriate antimicrobial treatment can significantly reduce mortality, morbidity, and hospital costs [8,9]. Rapid and accurate identification (ID) and antimicrobial susceptibility testing (AST) results of microorganisms from BSIs are critical prerequisites for the early selection of appropriate antimicrobial therapy and may lead to reductions in the misuse of broad-spectrum antibiotics, which are associated with greater adverse reactions and increased risk of bacterial resistance [10,11]. To this end, the Infectious Disease Society of America (IDSA) guidelines recommend the use of rapid diagnostic tests (RDTs) and their integration into antibiotic stewardship programs [12]. However, improving the methods for performing microbial ID and AST for BSIs to shorten the reporting time remains a substantial challenge facing microbiology laboratories worldwide.

Some rapid molecular-based species identification methods, such as DNA sequencing and pathogen-specific real-time polymerase chain reaction (PCR), have been developed in recent years, demonstrating faster and more accurate microbial species identification results. However, detecting with these methods is costly, time-consuming, and relies on special and expensive equipment, as well as higher technical expertise from the operators [13]. Therefore, the lack of fast, reliable, user-friendly, and cost-effective diagnostic methods is a significant limitation in managing BSIs [14].

MALDI-TOF MS can provide reliable identification results for bacterial colonies grown on solid media within minutes, while the mean incubation time required for species-level identification is 5.9 h for Gram-positive cocci (GPC) and 2.0 h for Gram-negative bacteria (GNB) [15,16]. Therefore, MALDI-TOF MS could serve as a new method for directly identifying bacteria from positive BCs [17,18]. To further improve the rate of identification of pathogens from positive BCs, various sample preparation protocols have been developed to enable direct bacterial identification from positive BCs via MALDI-TOF MS without the need for additional subculture [19–24]. However, there is currently no consensus on how to enrich pathogens from positive BCs prior to detection.

Currently, the relatively mature AST is conducted using automated instruments, which rely on the use of pure individual bacterial colonies after inoculation and culture, but this process typically takes 2–3 days. The European Committee on Antimicrobial Susceptibility Testing (EUCAST) and the Clinical and Laboratory Standards Institute (CLSI) in the United States released rapid AST for positive blood cultures in 2019 and 2021, respectively. Based on this, some researchers have established routine laboratory procedures for positive BCs by integrating MALDI-TOF MS rapid ID and rapid AST, enabling earlier clinical treatment [22,25–28]. Furthermore, several new rapid AST methods have emerged, such as a novel rapid direct E-test AST, which can detect antibiotic resistance within 5–6 hours and has shown excellent performance in terms of categorical agreement and error rates [29]. These modified workflows are considered appropriate for routine clinical laboratory environments because they can reduce costs and manual hands-on time [30]. However, no unified standard or consensus has been reached regarding the implementation conditions and expected effects of these workflows across different laboratories. Therefore, we aimed to explore some methods based on MALDI-TOF MS and rapid AST from BSIs under conditions in our laboratory to shorten the reporting time for BSIs, avoid overuse of antimicrobials, and improve patient satisfaction with medical treatment.

## 2. Materials and methods

### 2.1. Clinical specimens and blood culture system

All patients admitted to Sun Yat-Sen University Cancer Center between 01/06/2022 and 31/12/2023 with positive BCs from aerobic culture bottles (FAs) or anaerobic culture bottles (FNs) according to the results of the BactAlert VIRTUO or BacTAlert 3D microbial detection system (bioMérieux, Marcy l’Etoile, France) were included in this study. Both the FAs and FNs from the same patient were included in this study, but we only analyzed the data of positive BCs showing monomicrobial growth. This study was approved by the Ethics Committee of Sun Yat-sen University Cancer Center (B2025-698–01). This study is retrospective, and the specimens used were residual samples collected after completion of clinical testing for the participants. All data were accessed by the research team on 16/10/2025. All data were fully anonymized during the data processing stage. All identifiable information was permanently removed from the final dataset, which was used for all analyses and reporting in this manuscript.

### 2.2. AST system

AST was performed on a VITEK 2 fully automatic microbial identification and antimicrobial sensitivity analyzer system (bioMérieux, Marcy l’Etoile, France) with the accompanying antimicrobial sensitivity cards. The antimicrobial disks used in the Kirby-Bauer disc diffusion method were obtained from the OXOID Company (England).

### 2.3. Quality control strains

All quality control strains (*Escherichia coli* ATCC 25922/35218/8739, *Staphylococcus aureus* ATCC 25923/29213, *Enterococcus faecalis ATCC 29212*, *Klebsiella pneumoniae* ATCC 700603, *Pseudomonas aeruginosa* ATCC 27853, *Streptococcus pneumoniae* ATCC 49619, *Candida albicans* ATCC 14053, *Candida krusei* ATCC 6258, *Candida parapsilosis* ATCC 22019) came from the National Center for Clinical Laboratories (NCCL), and laboratory quality control was conducted once a month.

### 2.4. MALDI-TOF MS

The individual pathogenic microorganism colony incubated for 18–24 h or the pathogen obtained by pretreatment was scraped and smeared on the stainless steel target plate. On-plate extraction of Gram-positive bacteria (GPB) and fungi was performed by dissolving the cell walls with 0.7 μL of formic acid solution, allowing the target plate to air dry. Then 0.5 μL of CHCA (α-cyano-4-hydroxycinnamic acid) matrix was applied to each sample, which was air-dried prior to MALDI-TOF MS (bioMe´rieux, Marcy l’Étoile, France) analysis.

### 2.5. Conventional ID and AST methods

**Fig 1:**
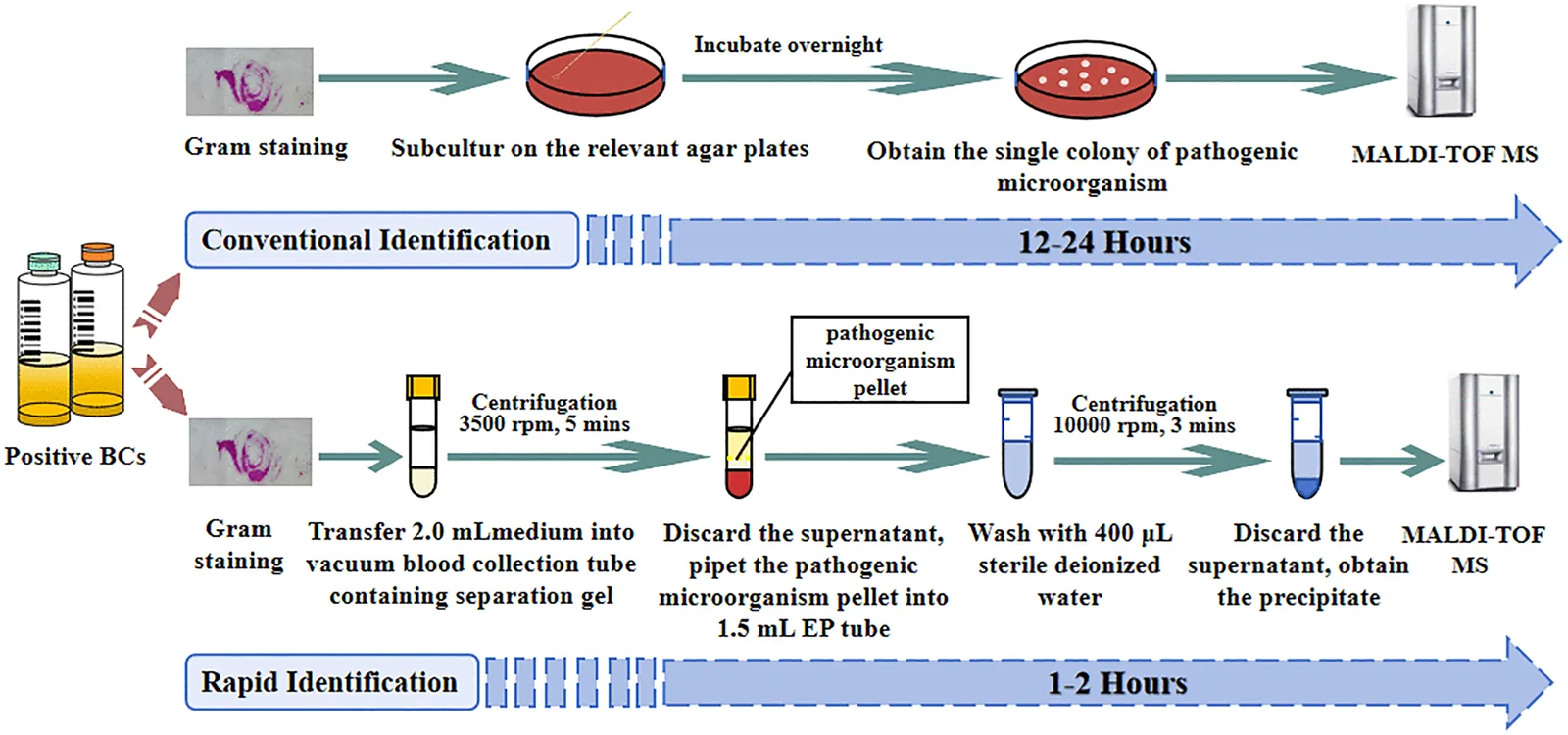
Workflow for conventional and rapid ID of pathogens from positive BCs. BC: blood culture; ID: identification; MALDI-TOF MS: matrix-assisted laser desorption/ionization time-of-flight mass spectrometry.

**Fig 2:**
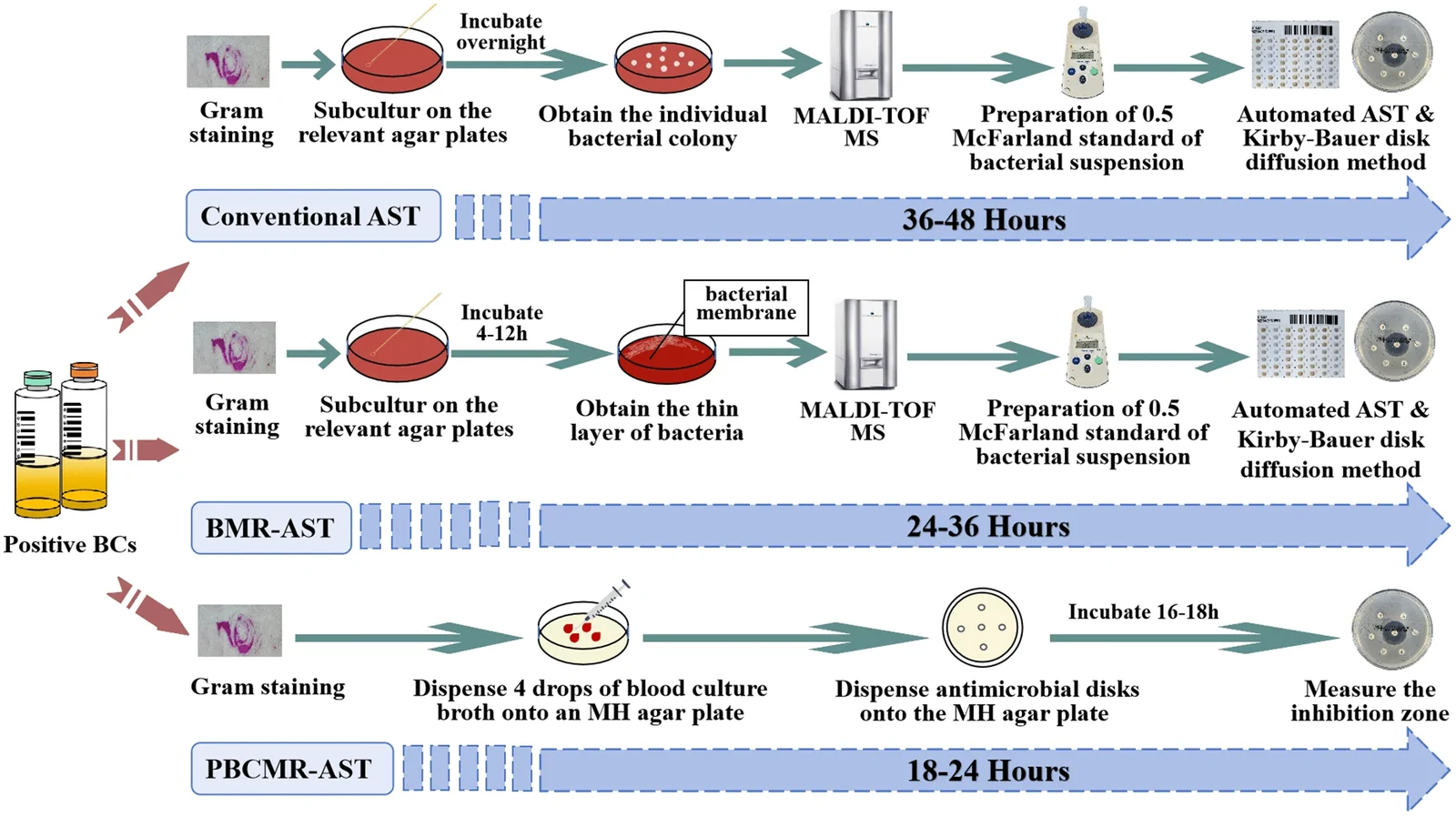
Workflow for conventional and rapid AST of pathogens from positive BCs. BC: blood culture; AST: antimicrobial susceptibility testing; BMR-AST: bacterial membrane rapid AST; PBCMR-AST: positive blood culture medium rapid AST; MALDI-TOF MS: matrix-assisted laser desorption/ionization time-of-flight mass spectrometry.

Positive BC medium was extracted for Gram staining and subcultured on relevant agar plates. The plates were then incubated overnight in an appropriate environment, and the individual pathogenic microorganism was identified by MALDI-TOF MS on the morning of the next day (Fig 1). Two or three individual colonies were then selected to prepare a bacterial suspension at a concentration equivalent to 0.5 McFarland standard, and the corresponding antimicrobial sensitivity card was selected for AST using the VITEK 2 system (Fig 2). In addition, we added some antimicrobial disks for the Kirby-Bauer disk diffusion method according to the requirements of the China Antimicrobial Resistance Surveillance System (CARSS) technical plan (2022). Owing to staffing constraints, we usually send the final ID and AST reports on the morning of the third day.

### 2.6. Rapid identification method

A total of 2.0 mL of positive BC medium was transferred into a vacuum blood collection tube containing separation gel (Fig 1). After centrifugation at 3,500 rpm at room temperature for 5 minutes, the blood components were located under the gel, and the pathogenic microorganisms were adhered to the gel, yielding a gelatinous membrane layer. Discard the supernatant liquid slowly. After carefully pipetting the residual serum from the precipitate, transfer the sediment on the gel surface into a 1.5 mL EP tube. The pellet was then washed with 400 μL sterile deionized water and centrifuged at 10,000 rpm for 3 minutes at room temperature. The supernatant was removed by pipetting, and the precipitate was smeared on the target plate for MALDI-TOF MS analysis. This method was generally performed within half an hour.

### 2.7. Bacterial membrane rapid AST (BMR-AST)

Gram staining and inoculation of positive BCs were performed as described above. The plates were incubated in a 5% carbon dioxide temperature chamber at 35℃ for 4–12 h, depending on the type of microorganism being identified. When a thin layer of bacterial colonies grew on the first region of the agar plate, the isolates were identified using MALDI-TOF MS. Then the AST was conducted using the VITEK 2 or the Kirby-Bauer disk diffusion method (Fig 2).

### 2.8. Positive blood culture medium rapid AST (PBCMR-AST)

Gram staining of positive BC samples was performed as described above. The positive BC bottles were inverted 5–10 times for thorough mixing. An appropriate amount of medium (approximately 100 μL) was inoculated onto a blood agar plate to detect the purity of the growing colonies. Another four drops of BC broth were added to MH agar plate, which was then evenly inoculated with a sterile cotton swab, after which the corresponding antimicrobial susceptibility disks were dispensed onto the surface of the inoculated MH agar plate (Fig 2). The plates were placed upside down in an incubator at 35℃ for 16–18 hours. Finally, the diameter of the inhibition zone(s) was measured, which is the basis for subsequently judging the drug sensitivity of the tested bacteria according to the corresponding standard.

### 2.9. Data analysis

In terms of ID, according to the MALDI-TOF MS system’s interpretation, organisms with a reliability > 90% were accepted as being correctly identified. The identification results were divided into three categories: not identified, misidentified, and full consistency. In terms of BMR-AST and PBCMR-AST, the minimum inhibitory concentration (MIC) values of the bacteria against the antimicrobials evaluated by both the rapid and conventional methods were translated into clinical categories (susceptible [S], intermediate [I], resistant [R], or susceptible-dosedependent [SDD]) according to Performance Standards for Antimicrobial Susceptibility Testing (CLSI M100 2023). Regarding the Kirby-Bauer disk diffusion method, the operation process and the breakpoints for the diameter of the inhibition zone (mm) were also defined according to CLSI M100.

For comparison of the results of these methods, the CLSI M52 2020 (Verification of Commercial Microbial Identification and Antimicrobial Susceptibility Testing System) and the ISO 20776–2:2021 (Clinical laboratory testing and in vitro diagnostic test systems-Susceptibility testing of infectious agents and evaluation of performance of antimicrobial susceptibility test devices-Part 2: Evaluation of performance of antimicrobial susceptibility test devices against reference broth micro-dilution) were used. In terms of rapid AST, the category agreements (CAs, the concordance of AST results compared with the reference method or the comparison method) required by WS/T 807–2022 (Recommended Health Industry Standard of China), the essential agreements (EAs, MIC results obtained from the AST device that fall within plus or minus one two-fold dilution of the MIC value established by the reference method or comparison method), the bias (evaluation of tested MIC results to determine whether the results that differ from the reference method are significantly skewed or predominantly in one direction), and the error rates were calculated [14]. Bias was computed from geometric mean MICs of test and reference methods. Bias > 0: The test method yielded higher MICs overall. Bias < 0: The test method yielded lower MICs overall. It is worth noting that the calculations of EAs and the biases are only applicable when MIC values are obtained using instrumental methods and are not applicable to the Kirby-Bauer disk diffusion method. The corresponding reference ranges and confidence intervals are listed in Table 1. Discrepancies were classified as major errors (MEs: false resistance, considered as resistant susceptibility by one method but susceptible by the other) and very major errors (VMEs: false susceptibility).

**Table 1:** Criteria for evaluating the concordance and error rates of AST.

Category	Rapid method	Conventional method	Acceptable standard
CA (category agreement)	S	S	≥ 90.0%
	R	R	
	I	I	
EA (essential agreement)	/	/	≥ 90.0%
Bias	/	/	−30% ~ +30%
ME (major error)	R	S	≤ 3.0%
VME (very major error)	S	R	≤ 3.0%

Basic numerical calculations were performed using Microsoft Excel. Graphs were plotted using GraphPad Prism. In this study, only descriptive analyses were conducted to evaluate the performance of rapid ID and AST across diverse pathogens, aiming to provide evidence for the subsequent optimization of laboratory protocols. Intergroup comparative statistical analyses were not predefined as primary endpoints in the original research design. Accordingly, only descriptive statistical parameters were calculated for all collected data, and intergroup statistical comparison tests were not conducted.

## 3. Results

### 3.1. Concordance between rapid and conventional identification methods

A total of 210 positive BCs were studied, including 118 Gram-negative bacteria (GNB), 78 Gram-positive bacteria (GPB), and 14 fungi. Sample IDs, blood-culture bottle types, time-to-positivity, and microbial identification results obtained by both rapid and conventional methods for all samples have been compiled into a table, as shown in Supplementary File 1. The identification concordance rate (ICR) was calculated using the MALDI-TOF MS results as the standard.

The ICR for GNB was 74.6% (88/118) (Table 2). A total of 26 strains were not identified (16 *E. coli*, 4 *E. cloacae*, 2 *E. asburiae*, 1 *P. aeruginosa*, 1 *Salmonella enterica* subsp. *enterica*, 1 *Acinetobacter junii*, 1 *Bacteroides fragilis*), and 4 strains were incorrectly identified, all of which were non-fermentative bacteria (2 *Acinetobacter pittii*, 1 *Aeromonas hydrophila*, 1 *Pseudomonas oryzihabitans*). Among GNB, fermentative isolates accounted for the majority (87.4%) with an ICR of 77.7% (80/103), which was superior to that for non-fermentative isolates (53.5%, 7/13). In addition, one of the two strains of anaerobic bacteria was correctly identified. We studied 78 GPB strains, and the ICR was only 37.2% (29/78) (Table 3). The 49 strains that could not be identified included 38 *Staphylococcus* spp., 4 *Enterococcus* spp., and 7 *Streptococcus* spp. *Staphylococcus* spp. constituted the majority of GPB species (67.9%), but the ICR was only 28.3% (15/53), which was much lower than that of *Enterococcus* spp. (69.2%, 9/13), followed by *Streptococcus* spp. (36.4%, 4/11). In addition, we also studied 14 strains of fungi (13 *Candida* and 1 *Cryptococcus*), but only 2 *C.albicans* were identified correctly, and the ICR was only 14.3% (2/14) (Table 4).

**Table 2:** ICRs for GNB in rapid identification method.

Organism	Rapid method	Conventional method	ICR
**Fermentative Bacteria**	**80**	**103**	**77.7%**
*Escherichia coli*	46	62	74.2%
*Enterobacter cloacae*	12	16	75.0%
*Klebsiella pneumoniae*	15	15	100.0%
*Enterobacter aerogenes*	4	4	100.0%
*Citrobacter koseri*	2	2	100.0%
*Salmonella enterica* subsp. *enterica*	1	2	50.0%
*Enterobacter asburiae*	0	2	0.0%
**Non-fermentative Bacteria**	**7**	**13**	**53.5%**
*Pseudomonas aeruginosa*	5	6	83.3%
*Aeromonas sobria*	1	1	100.0%
*Stenotrophomonas maltophilia*	1	1	100.0%
*Acinetobacter junii*	0	1	0.0%
*Acinetobacter pittii*	incorrectly identified	2	0.0%
*Aeromonas hydrophila*	incorrectly identified	1	0.0%
*Pseudomonas oryzihabitans*	incorrectly identified	1	0.0%
**Anaerobic GNB**	**1**	**2**	**50.0%**
*Bacteroides pyogenes*	1	1	100.0%
*Bacteroides fragilis*	0	1	0.0%
**Total**	**88**	**118**	**74.6%**

**Table 3:** ICRs for GPB in rapid identification method.

Organism	Rapid method	Conventional method	ICR
***Staphylococcus* spp.**	**15**	**53**	**28.3%**
*Staphylococcus aureus*	11	35	31.4%
*Staphylococcus epidermidis*	2	6	33.3%
*Staphylococcus hominis*	1	7	14.3%
*Staphylococcus haemolyticus*	1	1	100.0%
*Staphylococcus cohnii* subsp. *cohnii*	0	3	0.0%
*Staphylococcus caprae*	0	1	0.0%
***Enterococcus* spp.**	**9**	**13**	**69.2%**
*Enterococcus faecalis*	6	6	100.0%
*Enterococcus avium*	2	3	66.7%
*Enterococcus gallinarum*	1	2	50.0%
*Enterococcus faecium*	0	2	0.0%
***Streptococcus* spp.**	**4**	**11**	**36.4%**
*Streptococcus anginosus*	2	3	66.7%
*Streptococcus agalactiae*	2	2	100.0%
*Streptococcus constellatus* spp.	0	4	0.0%
*Streptococcus gallolyticus* ssp.	0	1	0.0%
*Streptococcus mitis/Streptococcus oralis*	0	1	0.0%
**Other GPB**	**1**	**1**	**100%**
*Lactobacillus casei*	1	1	100.0%
**Total**	**29**	**78**	**37.2%**

**Table 4:** ICRs for fungi in rapid identification method.

Organism	Rapid method	Conventional method	ICR
*Candida albicans*	2	8	25%
*Candida tropicalis*	0	4	0%
*Candida parapsilosis*	0	1	0%
*Cryptococcus neoformans*	0	1	0%
**Total**	**2**	**14**	**14.3%**

**Fig 3:**
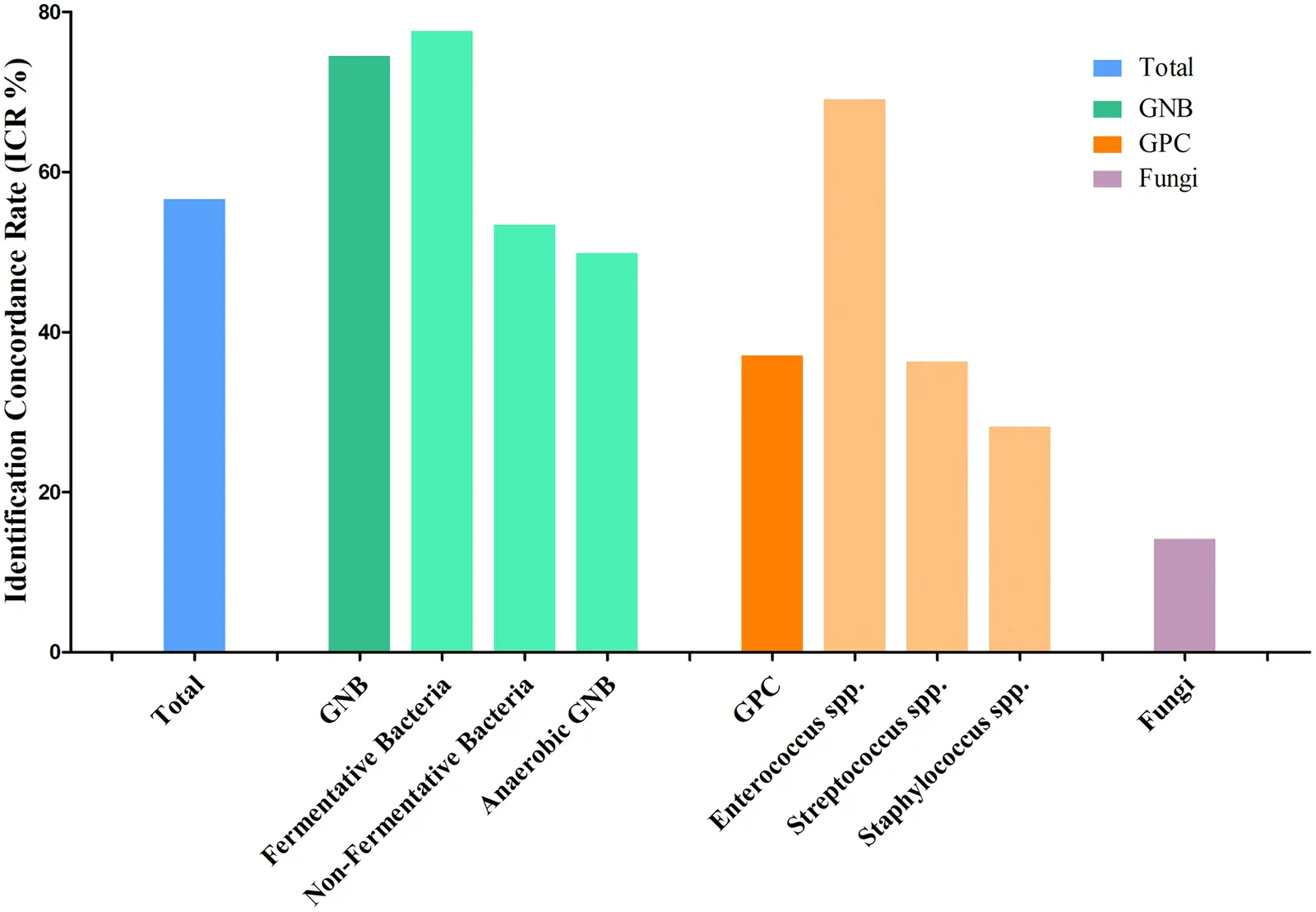
ICRs for rapid ID of pathogens from positive BCs. ICR: identification concordance rate; ID: identification; BC: blood culture; GNB: Gram-negative bacteria; GPC: Gram-positive cocci.

The ICRs for all positive BCs are shown in Fig 3. The ICR for GNB, especially that for fermentative bacteria, was much higher than that for GPC and fungi, suggesting that this rapid identification method is particularly suitable for the identification of GNB and especially for *Enterobacterales*.

We further analyzed the differences in ICRs between GNB and GPC in different BC bottles (Table 5). The data demonstrated that the ICRs for GNB were higher than those for GPC in both FAs (75.4% vs. 23.5%) and FNs (73.1% vs. 47.6%), which was consistent with our previous research results. There was no significant difference in ICRs for GNB between FAs and FNs (75.4% vs. 73.1%). Notably, FNs yielded significantly higher ICRs for GPC than FAs (47.6% vs. 23.5%). Overall, GNB detection was predominant in both bottles, while FNs exhibited a substantially superior detection capacity for GPC.

**Table 5:** ICRs for GNB and GPC in rapid identification method across different BC bottles.

FA/FN	Organism	Rapid method	Conventional method	ICRs
FA	GNB	46	61	75.4 %
	GPC	8	34	23.5 %
FN	GNB	38	52	73.1 %
	GPC	20	42	47.6 %

### 3.2. Concordance between BMR-AST and conventional AST method

A total of 82 isolates were investigated in this part, including 58 GNB and 24 GPC. For GNB, 55 fermentative bacteria (37 *Escherichia coli,* 8 *Klebsiella pneumoniae,* 6 *Enterobacter cloacae,* 2 *Citrobacter koseri,* 1 *Enterobacterium hominis,* 1 *Bacillus proteus*) and 3 non-fermentative bacteria (1 *Pseudomonas aeruginosa,* 1 *Acinetobacter baumannii, 1 Acinetobacter nosocomialis*) were studied. The results revealed that the CAs for all drugs exceeded 90% (mean 96.8%, 1125/1162) (Table 6). All EAs exceeded 90%, with a mean of 97.1%. In addition, all MIC biases fell within acceptable limits. In terms of error rates, MEs were detected in sulfamethoxazole/trimethoprim, cefoxitin, ceftazidime, ceftriaxone, cefuroxime, and cefuroxime axetil, while VMEs were detected in cefoperazone/sulbactam, cefepime, and ceftriaxone, but the error rates for all drugs were within the required range, except for cefoxitin (4.0%), suggesting that attention should be paid when using cephalosporin and cephalosporin compound formulation drugs. Regarding GPC, 22 *Staphylococcus* spp. and 2 *Enterococcus* spp. were investigated*.* The findings revealed that the CAs (mean 97.7%, 334/342) and EAs (mean 97.2%) for all tested antimicrobial agents exceeded 90%, with all MIC biases falling within acceptable limits (Table 7). Only one isolate of *Staphylococcus hominis* exhibited methicillin resistance, yielding an error rate of 4.5%. This discrepancy may be attributed to the limited sample size of this species. Notably, no VMEs were detected throughout this study.

**Table 6:** Analysis of CAs, EAs, bias, and error rates for GNB in BMR-AST [n (%)].

Antibiotics	n	VMEs	MEs	CAs	EAs	Bias
amikacin	59	0	0	59 (100)	59 (100)	0
cefoxitin	50	0	2 (4.0)	46 (92.0)	48 (96)	13.58
amoxicillin-clavulanic acid	48	0	0	44 (91.7)	45 (93.8)	−1.43
cefepime	59	1 (1.7)	0	54 (91.5)	55 (93.2)	−1.17
ceftazidime	59	0	1 (1.7)	56 (94.9)	57 (96.6)	16.41
ceftriaxone	55	1 (1.8)	1 (1.8)	53 (96.4)	52 (94.5)	−1.33
cefuroxime	55	0	1 (1.8)	53 (96.4)	53 (96.4)	7.85
ertapenem	55	0	0	55 (100)	55 (100)	−2.56
imipenem	59	0	0	58 (98.3)	58 (98.3)	−1.17
levofloxacin	59	0	0	56 (94.9)	58 (100)	−3.46
piperacillin/tazobactam	59	0	0	58 (98.3)	58 (98.3)	6.50
tigecycline	55	0	0	55 (100)	54 (98.2)	7.85
cefoperazone/sulbactam	59	1 (1.7)	0	58 (98.3)	58 (98.3)	−3.46
sulfamethoxazole/trimethoprim	55	0	1 (1.8)	54 (98.1)	54 (98.2)	5.17
cefuroxime axetil	55	0	1 (1.8)	53 (96.4)	53(96.4)	7.85
cefazolin	53	0	0	50 (94.3)	/	/
meropenem	57	0	0	57 (100)	/	/
ampicillin	52	0	0	52 (100)	/	/
aztreonam	55	0	0	53 (96.4)	/	/
ampicillin/sulbactam	51	0	0	48 (94.1)	/	/
gentamicin	53	0	0	53 (100)	/	/
**Total**	**1162**	**3 (0.3)**	**7 (0.6)**	**1125 (96.8)**	**97.1**	**3.38**

**Table 7:** Analysis of CAs, EAs, bias, and error rates for GPC in BMR-AST [n (%)].

Antibiotics	n	VMEs	MEs	CAs	EAs	Bias
moxifloxacin	22	0	0	22 (100)	21 (95.5)	6.21
oxacillin	22	0	1 (4.5)	21 (95.5)	21 (95.5)	13.43
vancomycin	24	0	0	24 (100)	24 (100)	5.70
linezolid	24	0	0	24 (100)	24 (100)	8.67
gentamicin	22	0	0	22 (100)	21 (95.5)	9.46
erythromycin	24	0	0	23 (95.8)	24 (100)	−2.73
teicoplanin	24	0	0	23 (95.8)	22 (91.7)	5.70
levofloxacin	24	0	0	22 (91.7)	23 (95.8)	8.32
rifampin	22	0	0	22 (100)	21 (95.5)	−6.02
tigecycline	24	0	0	24 (100)	24 (100)	−3.01
ceftaroline	18	0	0	18 (100)	18 (100)	0.00
clindamycin	22	0	0	20 (90.9)	21 (95.5)	16.26
Penicillin	24	0	0	24 (100)	24 (100)	0.00
daptomycin	24	0	0	23 (95.8)	23 (95.8)	−10.64
D-test	22	0	0	22 (100)	/	/
**Total**	**342**	**0**	**1 (0.3)**	**334 (97.7)**	**97.2**	**3.67**

### 3.3. Concordance between the PBCMR-AST and conventional AST methods

A total of 98 samples were investigated in this part of the study, including 70 GNB (50 *E. coli*, 9 *K. pneumoniae*, 6 *E. cloacae*, 2 *P. aeruginosa*, 1 *Acinetobacter baumannii complex*, 1 *Stenotrophomonas maltophilia*, and 1 *Aeromonas hydrophila)* and 28 GPC (16 *Staphylococcus aureus*, 5 *Streptococcus mitis / Streptococcus oralis*, 4 *Staphylococcus hominis*, 1 *Streptococcus constellatus*, 1 *Enterococcus faecalis,* and 4 other strains). Since the CLSI positive BC rapid AST is currently only applicable to *Enterobacterales* and *P. aeruginosa*, bacteria of other species were only investigated in an exploratory study. For 65 strains of *Enterobacterales*, the mean CA for all the drugs was 94.1% (270/287) (Table 8), validating the applicability of this rapid method for *Enterobacterales*. Only sulfamethoxazole/trimethoprim resulted in one case of VME, but the error rate was within an acceptable range. Notably, ceftazidime presented lower CA and higher ME. Due to the limited sample size, caution should be exercised when applying these reference values.

**Table 8:** Analysis of CAs and error rates for *Enterobacterales* in PBCMR-AST [n (%)].

Antibiotics	n	VMEs	MEs	CAs
aztreonam	63	0	1 (1.6)	61 (96.8)
ampicillin	60	0	0	59 (98.3)
ceftazidime	18	0	2 (11.1)	11 (61.1)
ceftriaxone	18	0	0	17 (94.4)
sulfamethoxazole/trimethoprim	63	1 (1.6)	0	62 (98.4)
meropenem	65	0	1 (1.5)	60 (92.3)
**Total**	**287**	**1 (0.3)**	**4 (1.4)**	**270 (94.1)**

In addition to CLSI, we also selected a broader range of GNB antimicrobial disks based on the CARSS Technical Plan (2022) and numerous clinical drug sensitivity reports. These included cefazolin, ampicillin/sulbactam, gentamicin, piperacillin/tazobactam, and levofloxacin. The results, which contained four strains of non-fermentative bacteria, are shown in Table 9. The data indicated that the mean CA for GNB was 88.2% (539/611), which was marginally lower than that of the drugs recommended by the CLSI. However, the CAs were greater than 90% for all drugs except ampicillin/sulbactam, piperacillin/tazobactam, ceftazidime, and cefazolin. MEs were detected for most antimicrobial agents; the error rates of ceftazidime and piperacillin/tazobactam even surpassed 3%. Among these agents, the reliability of ceftazidime results is relatively limited owing to the insufficient volume of relevant isolates. Piperacillin/tazobactam yielded a high proportion of SDD interpretations and demonstrated frequent interpretive discrepancies relative to the reference MIC method. Overall, the results obtained for both agents should be interpreted with caution. These findings demonstrate that this rapid AST method possesses promising clinical applicability. Nevertheless, studies with larger sample sizes and broader antimicrobial spectra are still required to further validate the performance of direct rapid AST from positive blood cultures.

**Table 9:** Analysis of CAs and error rates for GNB in PBCMR-AST [n (%)].

Antibiotics	n	VMEs	MEs	CAs
aztreonam	65	0	1 (1.5)	63 (97.0)
ampicillin	60	0	0	59 (98.3)
ceftazidime	18	0	2 (11.1)	11 (61.1)
ceftriaxone	18	0	0	17 (94.4)
sulfamethoxazole/trimethoprim	66	1 (1.5)	0	65 (98.5)
meropenem	66	0	1 (1.5)	61 (92.4)
gentamicin	62	0	1 (1.6)	61 (98.4)
cefazolin	62	0	1 (1.6)	53 (85.5)
levofloxacin	68	0	0	65 (95.6)
ampicillin/sulbactam	59	0	1 (1.7)	53 (89.8)
piperacillin/tazobactam	67	0	5 (7.4)	31 (46.3)
**Total**	**611**	**1 (0.2)**	**12 (1.9)**	**539 (88.2)**

To further expand direct rapid drug susceptibility testing for positive BCs and improve its applicability in clinical practice, we also studied 28 isolates of GPC using antimicrobial disks containing penicillin, vancomycin, linezolid, erythromycin, and clindamycin (Table 10). The results revealed that the mean CA for GPC was 86.2% (100/116), with CAs over 95% for penicillin, erythromycin, linezolid, and vancomycin. No VMEs were observed, but a single ME (3.6%) was seen for erythromycin. Notably, clindamycin showed a markedly elevated ME rate of 32.1%, which was attributed to the absence of D-test (erythromycin-clindamycin disk approximation test) validation in the rapid AST. A positive D-test confirms inducible clindamycin resistance and mandates a final report of resistant result regardless of the initial susceptibility result. Consequently, integrating the D-test is critical for improving the accuracy of future clindamycin susceptibility testing.

**Table 10:** Analysis of CAs and error rates for GPC in PBCMR-AST [n (%)].

Antibiotics	n	VMEs	MEs	CAs
penicillin	24	0	0	23 (95.8)
erythromycin	28	0	1 (3.6)	27 (96.4)
clindamycin	28	0	9 (32.1)	15 (53.6)
Linezolid	28	0	0	27 (96.4)
vancomycin	8	0	0	8 (100)
**Total**	**116**	**0**	**10** (**8.6**)	**100** (**86.2**)

In summary, significant statistical concordance existed between the two rapid AST assays upon removing data of low interpretive validity, which originated from inadequate isolate numbers, inconsistent readings under different breakpoints, and the lack of confirmatory testing (Table 11). These results indicate that PBCMR-AST for positive BCs holds great promising clinical applicability in the future. Nevertheless, larger-scale cohorts and expanded AST datasets are needed to further validate its overall performance.

**Table 11:** Analysis of CAs and error rates for GNB and GPC in PBCMR-AST [n (%)].

Antibiotics	n	VMEs	MEs	CAs
aztreonam	65	0	1 (1.5)	63 (97.0)
ampicillin	60	0	0	59 (98.3)
sulfamethoxazole/trimethoprim	66	1 (1.5)	0	65 (98.5)
meropenem	66	0	1 (1.5)	61 (92.4)
gentamicin	62	0	1 (1.6)	61 (98.4)
cefazolin	62	0	1 (1.6)	53 (85.5)
levofloxacin	68	0	0	65 (95.6)
ampicillin/sulbactam	59	0	1 (1.7)	53 (89.8)
penicillin	24	0	0	23 (95.8)
erythromycin	28	0	1 (3.6)	27 (96.4)
linezolid	28	0	0	27 (96.4)
**Total**	**588**	**1 (0.2)**	**6 (1.0)**	**557 (94.7)**

**Fig 4:**
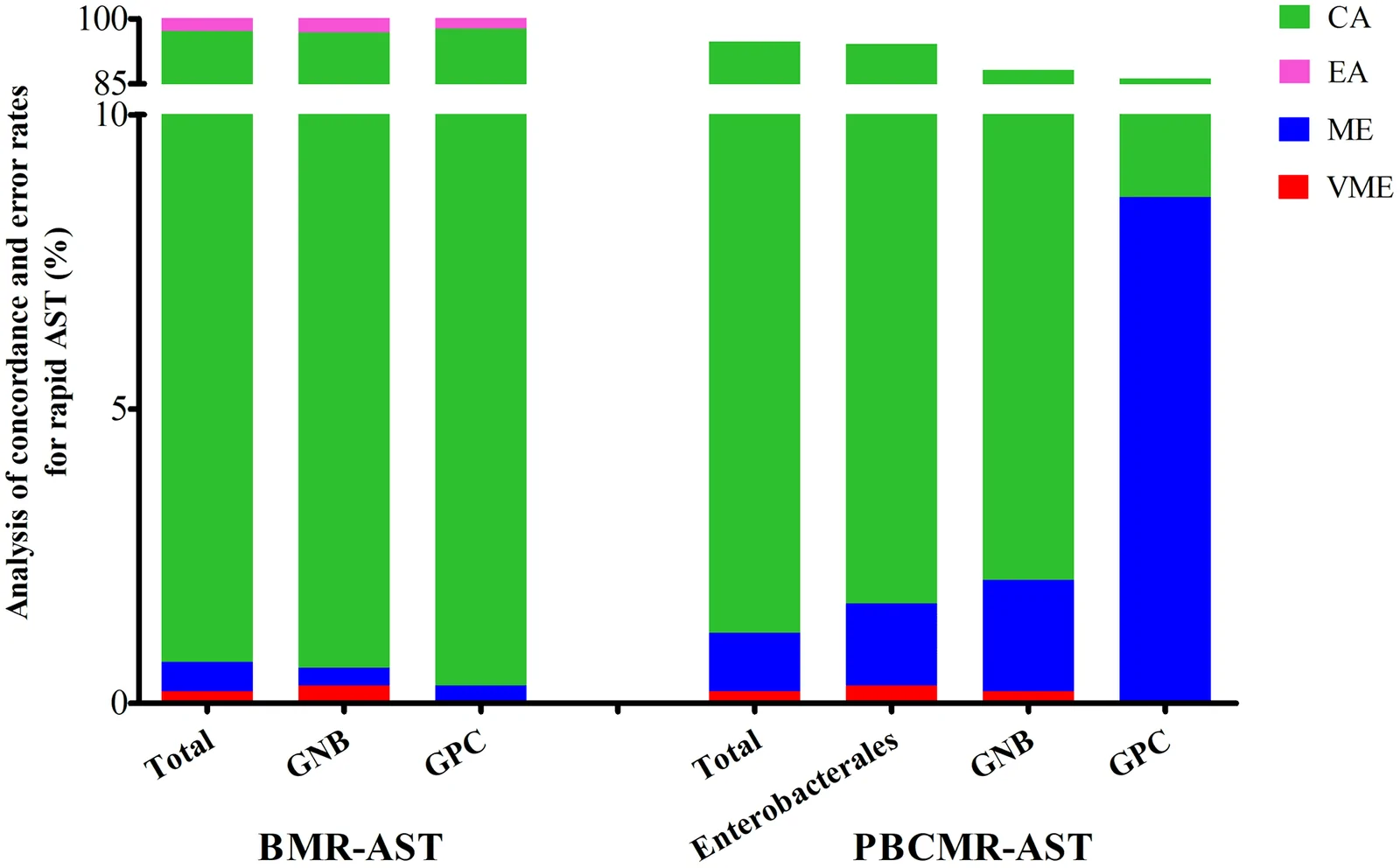
Analysis of concordance and error rates for rapid AST of pathogens from positive BCs. BC: blood culture; AST: antimicrobial susceptibility testing; BMR-AST: bacterial membrane rapid AST; PBCMR-AST: positive blood culture medium rapid AST; GNB: Gram-negative bacteria; GPC: Gram-positive cocci; CA: category agreement; EA: essential agreement; ME: major error; VME: very major error.

Finally, we summarized the concordance and error rates between the two rapid AST methods and the conventional method (Fig 4).

## 4. Discussion

Timely administration of appropriate antimicrobials constitutes a critical factor for lowering morbidity and mortality among patients with BSIs. Rapid, accurate pathogen ID and AST are therefore indispensable, particularly for pathogens outside the coverage of standard empirical antimicrobial regimens. However, traditional blood subculture approaches are time-consuming. Microbes grow on solid media within 24–48 h, and ID/AST takes another 24–48 h, meaning that clinicians need to wait 48–96 h for full reports after the BCs show positive [29]. This delay not only cannot meet clinical needs, but also increases the risk of doctor-patient conflicts. Our research has demonstrated that the combination of rapid ID and AST can shorten the overall detection period by 24–48 h. This protocol relies on non-culture or short-incubation assays, so the reduced turnaround time refers to the duration from inoculation to single-colony formation for most pathogens, based on the long-term experience in our lab. Since pathogen species, microbial load, and growth rate all affect the detection timeline, we report time savings as a range instead of a fixed value.

Currently, several new technologies are available for direct ID and AST from whole blood. However, their widespread deployment in clinical microbiology laboratories is constrained by limitations in institutional classification, infrastructure capacity, cost management, and workforce expertise. We previously performed a multicenter study that focused mainly on large tertiary hospitals in Guangzhou, China. Notably, even these high-level institutions currently lack the qualified conditions required for direct rapid pathogen ID and AST from blood specimens [31]. Blood culture remains the gold standard and first-line tool for identifying the pathogens responsible for BSIs. Therefore, establishing an optimal workflow for direct rapid ID and AST using positive BC broth without prior subculture represents a critical priority for most contemporary clinical microbiology laboratories.

Multiple clinical studies have explored and validated rapid identification methods for positive BCs, which predominantly rely on MALDI-TOF MS after specimen preprocessing. However, this pretreatment often requires tedious manual steps to obtain sufficient and purified pathogen proteins [32]. As hemoglobin, activated carbon, or resin within BC bottles can disrupt protein spectra and compromise the identification results, pathogen enrichment and purification through pretreatment are indispensable for rapid ID. Existing pretreatment methods can be divided into kit-based extraction methods, blood cell lysis methods, differential centrifugation methods, and separation gel methods [20,25,33–35]. This study evaluated direct MALDI-TOF MS identification of positive BCs using gel separation vacuum tubes.

In our study, the ICRs for GNB, GPB, and fungi were 74.6%, 37.2%, and 14.3%, respectively. The ICRs for GNB were significantly higher than those for GPB and fungi, possibly due to the slower growth of GPB and the thick cell walls of fungi, which make protein extraction more difficult [19,22,36,37]. And among GNB, the ICR for fermentative bacteria was much higher than that for non-fermentative bacteria (77.7% vs. 53.5%), which may be related to the fast reproduction of *Enterobacterales* bacteria. These findings are in good agreement with those from previous domestic and international studies. According to the literature, the species-level identification accuracy of direct MALDI-TOF MS analysis of positive BC broths ranges from 50.5% to 93.2%, which varies by isolate composition, pretreatment/extraction method, and the definitions of the cutoff threshold log scores [20,22,32,38–47]. Azrad M et al. adopted this approach for 186 samples and documented an overall ICR of 45.5%, of which 52% was for GNB and 43% for GPB [20]. Zengin-Canalp H et al. analyzed 295 specimens and reported an overall ICR of 93.2%, with 100% for GNB and 88.9% for GPB [22]. Kuo SF et al. analyzed 354 samples and reached an overall ICR of 63.3%, with 88.2% for GNB and 49.7% for GPB [45]. Ashmawy MM et al. investigated 128 specimens and obtained an overall ICR of 75.8%, including 90.16% for GNB, 69.1% for GPB, and 33.3% for yeasts [46]. Matúšková LS et al. reported an overall ICR of 74.81% among 135 specimens, with ICRs of 93.05% for GNB and 53.97% for GPB [47]. The considerable variation in overall ICR across these studies may be attributed to multiple factors. First, differences in the proportion of GNB, GPB, and fungi among the enrolled specimens could largely influence the pooled performance, given that GNB generally yield substantially higher ICRs. Second, variations in sample pre-processing protocols, centrifugation-washing conditions, and the blood-culture bottle types may also introduce inter-study discrepancies. In addition, different MALDI-TOF MS score thresholds adopted by each research group further contribute to inconsistent outcomes. Collectively, these observations highlight that the performance of direct MALDI-TOF MS from positive blood culture broths is highly context-dependent, and local laboratory validation is indispensable before routine clinical implementation.

We further analyzed how BC bottle types affect ICR. In both bottles, GNB yielded higher ICR than GPC, but the ICR for GNB showed no inter-bottle difference (75.4% vs 73.1%), likely because of their fast proliferation yielding comparable bacterial loads and detection performance. In contrast, FNs achieved significantly higher ICR than FAs for GPC (47.6% vs. 23.5%), possibly because slow-growing GPC thrive better in FNs, which contain richer nutrients and have a stronger antibiotic adsorption capacity.

Prior studies on direct *Candida* spp. identification from positive BCs yield divergent results (0%–100%) due to varied sample pretreatment protocols [38,41,48–55]. However, small sample sizes in these studies hinder accurate ICR assessment. According to existing clinical experience, the detection of fungemia often requires an additional 24–48 hours of culture period compared with the detection of most clinically significant bacteremia, which supports the view that increasing broth volume and prolonging lysis time of positive BCs containing fungi can help improve the identification effect.

Notably, the ICR for *Staphylococcus* spp. was less than 30%, and coagulase-negative *Staphylococcus* (CoNS) showed a lower ICR than *Staphylococcus aureus* (22.2% vs. 31.4%). CoNS isolates frequently represent contamination, so this distinction may be key to clarifying the etiology of BSIs, as it allows the rapid distinction between true pathogens and possible contaminants [14,56]. We believe that further research on this topic will help distinguish pathogenic and non-pathogenic microorganisms. In particular, we believe that unnecessary antibiotic treatment can be avoided for patients with GPC infections, because contaminants can be identified earlier in GPC infections than in GNB infections.

Once accurate ID results of microorganisms are obtained, fast and accurate AST can then guide clinical antimicrobial selection. Traditional AST usually takes 2–3 days from culture to confirm antibiotic susceptibility, which delays targeted therapy. However, initial empirical antibiotic treatments are sometimes inadequate because they are either ineffective or involve unnecessary broad-spectrum antimicrobial therapies [57]. To overcome these drawbacks, several attempts have been made to develop rapid and accurate AST methods to shorten AST results and decrease mortality rates [58]. In this study, we analyzed 82 positive BC samples via BMR-AST and 98 via PBCMR-AST. Both methods yielded relatively high CAs but exhibited varying error rates. The reasons for these incorrect results involve not only the inherent characteristics of microorganisms or antibacterial drugs themselves, but also differences in bacterial growth cycles among different isolates, which may lead to the emergence of heterogeneous drug resistance. A previous study concluded that these bacteria are indeed resistant to a specific antibiotic, but the results might be incorrectly reported as susceptible if the bacteria are in the lag phase of their growth cycles [57].

In the BMR-AST test, a total of 58 GNB and 24 GPC were isolated. The mean CAs were 96.8% and 97.7%, and the mean EAs were 97.1% and 97.2%, respectively. All bias values were within the confidence interval. MEs were observed for a subset of the tested isolates, with rates of 0.6% for GNB and 0.3% for GPC, and one VME with a rate of 0.3% was detected among GNB. Most errors (both MEs and VMEs) occurred in tests involving cephalosporin or cephalosporin-based combination agents, and the MIC values were relatively high. This may be attributed to the inherent biological characteristics of the bacterial isolates. These strains grow rapidly, enabling them to reach sufficient biomass within a short time frame. In addition, these isolates may show constitutive overproduction of β-lactamases, reduced outer membrane permeability, or upregulated efflux systems. These inherent resistance mechanisms act rapidly without requiring prolonged incubation, leading to higher overall MIC values. For PBCMR-AST, the mean CA was 87.6% among 70 GNB, and was 86.2% among 28 GPC. Although the results were lower than BMR-AST, our findings are consistent with domestic and global reports. The drugs with high error rates in GNB included ceftazidime and piperacillin/tazobactam. The high error rate for ceftazidime can be explained by its small sample size, while the K-B method produced more SDD results for piperacillin/tazobactam, leading to limited reference values for comparison. With the exception of clindamycin, which exhibited a high error rate attributable to the absence of D-test detection, the CAs for GPC were at a high level. Several direct rapid AST studies from positive BCs have revealed very good CAs for GNB but poorer CAs for GPC [59,60]. In our study, the CAs for GNB and GPC were similar, possibly due to the relatively small sample size of GPC and the limited number of antibiotic types tested in this study. With regard to rapid AST, most studies have focused on determining MICs using isolates from short-term cultured plates. Matúšková LS et al. observed that for 125 bacterial isolates, their rapid AST method achieved a CA of 99.31%, a minor error rate (mE) of 0.46%, and a major error rate (ME) of 0.23% [47]. When stratified by pathogen group, the CA reached 100% for GPB and 98.9% for GNB, with only 0.7% minor error and 0.4% major error observed among GNB. For 70 *Enterobacterales* isolates tested against 17 antimicrobial agents, the CA reached 98.82%, the minor error rate was 0.76% (observed in tests of ceftazidime, cefoperazone-sulbactam, cefepime, and ciprofloxacin), and the major error rate was 0.42% (observed in tests of tobramycin and ciprofloxacin). Liang B et al. performed pellet-based rapid AST on 112 isolates and obtained an overall CA of 95.5%, with both very major discrepancy (MD) and minor discrepancy (mD) below 3% [61]. The inconsistent performance of rapid AST across published studies can be explained by multiple factors. Variations in pre-incubation time, bacterial pellet purification procedures, and residual blood-derived contaminants all affect bacterial purity and metabolic activity, which can lead to shifts in MIC readings and changes in error rates. Differences in pathogen composition and regional resistance profiles among different study cohorts are also important contributors to inter-study inconsistencies. Specifically, fast-growing *Enterobacterales* tend to produce more consistent and credible rapid-AST results, while GPB are associated with a significantly higher probability of elevated error rates. Furthermore, various AST panels, inconsistent incubation conditions, non-uniform MIC interpretation criteria, and residual antibiotic carry-over from positive blood culture bottles can all further undermine the consistency of results. Therefore, laboratory-specific validation is mandatory prior to the clinical implementation of direct rapid-AST workflows in routine diagnostic practice.

CLSI M100 released a disk diffusion test for positive BC broths and published breakpoints for the interpretation of several drug sensitivities against *Enterobacterales*; therefore, we conducted a separate analysis of the PBCMR-AST results of *Enterobacterales* strains. For 65 strains of *Enterobacterales*, the mean CA for all the drugs reached 94.1%, and the ME was 1.4%. This result was more ideal than those obtained for GNB and GPC, indicating that PBCMR-AST may have high clinical value for the early management of GNB infections, particularly for BSIs caused by *Enterobacterales*.

However, this study has several limitations. (1) The number and variety of strains tested at the species level in this study may be insufficient, especially for rapid AST. Further research on a larger number of microbial species is needed. (2) This research is only applicable to BSIs caused by a single microorganism. AST cannot be performed for some specific drug resistance phenotypes, such as extended-spectrum beta-lactamase (ESBL)-producing bacteria, Methicillin-resistant *Staphylococcus aureus* (MRSA), and carbapenem-resistant organisms (CROs), which should be reported in routine reports. (3) This was a single-center research. Since different hospitals have different sets of data on bacterial profiles and antibiotic resistance, there may be some unknown factors that could affect the results. Therefore, multicenter studies are needed to verify the effectiveness of these methods. (4) Our study is currently limited to basic research conducted in the microbiology laboratory, and further validation in improvements in clinical practice, such as patient mortality, the duration and cost of hospital stays, and actual antibiotic usage, could not be evaluated. (5) Our research may only be applicable to most current microbiology laboratories that are currently unable to introduce advanced technology platforms such as next-generation sequencing (NGS) or digital PCR. Indeed, our study was conducted on the basis of the mature ID and AST technologies, focusing solely on the pretreatment of positive blood culture specimens and the subsequent optimization of laboratory workflows. Given that this study did not involve the development of novel detection technologies, the novelty of our findings has certain limitations.

## 5. Conclusion

In this study, we established an optimized workflow for rapid pathogen identification and AST using positive BCs. Combining vacuum separation-gel blood collection tubes with MALDI-TOF MS enabled reliable identification of GNB (particularly *Enterobacterales*) within half an hour, with no requirement for specialized additional equipment or extra consumable costs. In our dataset, however, this workflow exhibited sub-optimal performance for GPB and fungi. Accordingly, we recommend applying this method only following Gram staining interpretation to avoid diagnostic misjudgment and reduce unnecessary laboratory workload.

The two rapid AST workflows showed good concordance with the conventional method, yet they presented different practical performance trade-offs. BMR-AST achieved higher concordance rates and provided more accurate AST data to facilitate precise clinical therapy. In contrast, PBCMR-AST provided results with a shorter turnaround time, offering greater clinical value for guiding antimicrobial therapy in critically-ill patients. Both workflows are technically feasible for implementation in routine clinical microbiology laboratories and serve as valuable complementary alternatives to conventional AST.

The integrated rapid identification and AST strategy reduced the overall diagnostic turnaround time by 24–48 h relative to standard workflows, which could support early targeted antimicrobial therapy and strengthen antimicrobial stewardship. Nevertheless, diagnostic performance varied substantially among different microbial groups. Large-scale multicenter studies are therefore further needed to validate and extend its real-world clinical applicability.

## Supporting information

S1 File This document contains a table presenting anonymized sample information, including sample ID, time-to-positivity, blood-culture bottle type, Gram staining, and the microbial identification results obtained by both the rapid method and the conventional method. (XLSX)

## References

[1] Balk RA. Severe sepsis and septic shock. Definitions, epidemiology, and clinical manifestations. Crit Care Clin. 2000;16(2):179–92. doi: 10.1016/s0749-0704(05)70106-8 10768078

[2] Goto M, Al-Hasan MN. Overall burden of bloodstream infection and nosocomial bloodstream infection in North America and Europe. Clin Microbiol Infect. 2013;19(6):501–9. doi: 10.1111/1469-0691.12195 23473333

[3] Evans L, Rhodes A, Alhazzani W, Antonelli M, Coopersmith CM, French C, et al. Surviving sepsis campaign: international guidelines for management of sepsis and septic shock 2021. Intensive Care Med. 2021;47(11):1181–247. doi: 10.1007/s00134-021-06506-y 34599691 PMC8486643

[4] Castillo DJ, Rifkin RF, Cowan DA, Potgieter M. The Healthy Human Blood Microbiome: Fact or Fiction? Front Cell Infect Microbiol. 2019;9:148. doi: 10.3389/fcimb.2019.00148 31139578 PMC6519389

[5] Kumar A, Ellis P, Arabi Y, Roberts D, Light B, Parrillo JE, et al. Initiation of inappropriate antimicrobial therapy results in a fivefold reduction of survival in human septic shock. Chest. 2009;136(5):1237–48. doi: 10.1378/chest.09-0087 19696123

[6] Kim KJ, Yun SG, Cho Y, Nam M-H, Ko YJ, Lee CK. Evaluation of a sterile, filter-based, in-house method for rapid direct bacterial identification and antimicrobial susceptibility testing using positive blood culture. Eur J Clin Microbiol Infect Dis. 2023;42(6):691–700. doi: 10.1007/s10096-023-04592-y 37012540

[7] Maddalena C, Giuseppe M, Perez M, Guido S, Stefania S. Evaluation of the liquid colony for identification and antimicrobial susceptibility testing directly from positive blood cultures. Ann Clin Microbiol Antimicrob. 2023;22(1):72. doi: 10.1186/s12941-023-00617-8 37568240 PMC10422792

[8] Beekmann SE, Diekema DJ, Chapin KC, Doern GV. Effects of rapid detection of bloodstream infections on length of hospitalization and hospital charges. J Clin Microbiol. 2003;41(7):3119–25. doi: 10.1128/JCM.41.7.3119-3125.2003 12843051 PMC165359

[9] Bhatti MM, Boonlayangoor S, Beavis KG, Tesic V. Rapid identification of positive blood cultures by matrix-assisted laser desorption ionization-time of flight mass spectrometry using prewarmed agar plates. J Clin Microbiol. 2014;52(12):4334–8. doi: 10.1128/JCM.01788-14 25232166 PMC4313308

[10] Kang C-I, Kim S-H, Park WB, Lee K-D, Kim H-B, Kim E-C, et al. Bloodstream infections caused by antibiotic-resistant gram-negative bacilli: risk factors for mortality and impact of inappropriate initial antimicrobial therapy on outcome. Antimicrob Agents Chemother. 2005;49(2):760–6. doi: 10.1128/AAC.49.2.760-766.2005 15673761 PMC547233

[11] Kollef MH. Broad-spectrum antimicrobials and the treatment of serious bacterial infections: getting it right up front. Clin Infect Dis. 2008;47 Suppl 1:S3–13. doi: 10.1086/590061 18713047

[12] Barlam TF, Cosgrove SE, Abbo LM, MacDougall C, Schuetz AN, Septimus EJ, et al. Implementing an Antibiotic Stewardship Program: Guidelines by the Infectious Diseases Society of America and the Society for Healthcare Epidemiology of America. Clin Infect Dis. 2016;62(10):e51–77. doi: 10.1093/cid/ciw118 27080992 PMC5006285

[13] Altun O, Botero-Kleiven S, Carlsson S, Ullberg M, Özenci V. Rapid identification of bacteria from positive blood culture bottles by MALDI-TOF MS following short-term incubation on solid media. J Med Microbiol. 2015;64(11):1346–52. doi: 10.1099/jmm.0.000168 26361761

[14] Akgun S, Sayiner HS. Comparison of Rapid and Routine Methods of Identification and Antibiotic Susceptibility Testing of Microorganisms from Blood Culture Bottles. Pol J Microbiol. 2020;69(2):1–12. doi: 10.33073/pjm-2020-019 32412189 PMC7324860

[15] Idelevich EA, Schüle I, Grünastel B, Wüllenweber J, Peters G, Becker K. Rapid identification of microorganisms from positive blood cultures by MALDI-TOF mass spectrometry subsequent to very short-term incubation on solid medium. Clin Microbiol Infect. 2014;20(10):1001–6. doi: 10.1111/1469-0691.12640 24698361

[16] Kohlmann R, Hoffmann A, Geis G, Gatermann S. MALDI-TOF mass spectrometry following short incubation on a solid medium is a valuable tool for rapid pathogen identification from positive blood cultures. Int J Med Microbiol. 2015;305(4–5):469–79. doi: 10.1016/j.ijmm.2015.04.004 25953498

[17] Fothergill A, Kasinathan V, Hyman J, Walsh J, Drake T, Wang YFW. Rapid identification of bacteria and yeasts from positive-blood-culture bottles by using a lysis-filtration method and matrix-assisted laser desorption ionization-time of flight mass spectrum analysis with the SARAMIS database. J Clin Microbiol. 2013;51(3):805–9. doi: 10.1128/JCM.02326-12 23254131 PMC3592086

[18] Wüppenhorst N, Consoir C, Lörch D, Schneider C. Direct identification of bacteria from charcoal-containing blood culture bottles using matrix-assisted laser desorption/ionisation time-of-flight mass spectrometry. Eur J Clin Microbiol Infect Dis. 2012;31(10):2843–50. doi: 10.1007/s10096-012-1638-2 22639175

[19] Kayin M, Mert B, Aydemir S, Özenci V. Comparison of rapid BACpro® II, Sepsityper® kit and in-house preparation methods for direct identification of bacteria from blood cultures by MALDI-TOF MS with and without Sepsityper® module analysis. Eur J Clin Microbiol Infect Dis. 2019;38(11):2133–43. doi: 10.1007/s10096-019-03654-4 31494828 PMC6800852

[20] Azrad M, Keness Y, Nitzan O, Pastukh N, Tkhawkho L, Freidus V, et al. Cheap and rapid in-house method for direct identification of positive blood cultures by MALDI-TOF MS technology. BMC Infect Dis. 2019;19(1):72. doi: 10.1186/s12879-019-3709-9 30658585 PMC6339441

[21] Tsuchida S, Murata S, Miyabe A, Satoh M, Takiwaki M, Matsushita K, et al. An in-house centrifugation and membrane filtration technique for identifying microorganisms from positive blood culture bottles with high identification rates using matrix-assisted laser desorption ionization-Time-of-flight mass spectrometry: A preliminary report. J Infect Chemother. 2020;26(3):266–71. doi: 10.1016/j.jiac.2019.09.017 31678054

[22] Zengin Canalp H, Bayraktar B. Direct Rapid Identification from Positive Blood Cultures by MALDI-TOF MS: Specific Focus on Turnaround Times. Microbiol Spectr. 2021;9(3):e0110321. doi: 10.1128/spectrum.01103-21 34908465 PMC8672911

[23] Zhou M, Yang Q, Kudinha T, Sun L, Zhang R, Liu C, et al. An Improved In-house MALDI-TOF MS Protocol for Direct Cost-Effective Identification of Pathogens from Blood Cultures. Front Microbiol. 2017;8:1824. doi: 10.3389/fmicb.2017.01824 29033904 PMC5625089

[24] Oviaño M, Ingebretsen A, Steffensen AK, Croxatto A, Prod’hom G, Quiroga L, et al. Multicenter Evaluation of Rapid BACpro® II for the Accurate Identification of Microorganisms Directly from Blood Cultures Using MALDI-TOF MS. Diagnostics. 2021;11(12):2251. doi: 10.3390/diagnostics11122251 PMC8700617 34943488

[25] Cruz S, Abreu D, Gomes R, Martins-Oliveira I, Silva-Dias A, Perez-Viso B, et al. An improved protocol for bacteria identification by MALDI-TOF MS directly from positive blood cultures. Eur J Clin Microbiol Infect Dis. 2024;43(3):605–10. doi: 10.1007/s10096-023-04725-3 38112967 PMC10917851

[26] Vaishnav MS, Pd D, Hegadi SS, C D, Murthy KNC, Srikanta S, et al. Accelerated microbial identification “directly” from positive blood cultures using MALDI-TOF MS: Local clinical laboratory challenges. Diagn Microbiol Infect Dis. 2024;109(3):116306. doi: 10.1016/j.diagmicrobio.2024.116306 38735146

[27] Dai Y, Xu X, Yan X, Li D, Cao W, Tang L, et al. Evaluation of a Rapid and Simplified Protocol for Direct Identification of Microorganisms From Positive Blood Cultures by Using Matrix Assisted Laser Desorption Ionization Time-of-Flight Mass Spectrometry (MALDI-TOF MS). Front Cell Infect Microbiol. 2021;11:632679. doi: 10.3389/fcimb.2021.632679 33777845 PMC7990877

[28] Kumar M, Shergill SPS, Tandel K, Sahai K, Gupta RM. Direct antimicrobial susceptibility testing from positive blood culture bottles in laboratories lacking automated antimicrobial susceptibility testing systems. Med J Armed Forces India. 2019;75(4):450–7. doi: 10.1016/j.mjafi.2018.08.010 31719741 PMC6838483

[29] Bianco G, Iannaccone M, Boattini M, Cavallo R, Costa C. Assessment of rapid direct E-test on positive blood culture for same-day antimicrobial susceptibility. Braz J Microbiol. 2019;50(4):953–9. doi: 10.1007/s42770-019-00139-6 31432464 PMC6863220

[30] Verroken A, Defourny L, Lechgar L, Magnette A, Delmée M, Glupczynski Y. Reducing time to identification of positive blood cultures with MALDI-TOF MS analysis after a 5-h subculture. Eur J Clin Microbiol Infect Dis. 2015;34(2):405–13. doi: 10.1007/s10096-014-2242-4 25252627

[31] Liu W, Liao K, Wu J, Liu S, Zheng X, Wen W, et al. Blood culture quality and turnaround time of clinical microbiology laboratories in Chinese Teaching Hospitals: A multicenter study. J Clin Lab Anal. 2024;38(1–2):e25008. doi: 10.1002/jcla.25008 38235610 PMC10829685

[32] Romero-Gómez M-P, Gómez-Gil R, Paño-Pardo JR, Mingorance J. Identification and susceptibility testing of microorganism by direct inoculation from positive blood culture bottles by combining MALDI-TOF and Vitek-2 Compact is rapid and effective. J Infect. 2012;65(6):513–20. doi: 10.1016/j.jinf.2012.08.013 22940580

[33] Barnini S, Brucculeri V, Morici P, Ghelardi E, Florio W, Lupetti A. A new rapid method for direct antimicrobial susceptibility testing of bacteria from positive blood cultures. BMC Microbiol. 2016;16(1):185. doi: 10.1186/s12866-016-0805-5 27520338 PMC4982226

[34] Caspar Y, Garnaud C, Raykova M, Bailly S, Bidart M, Maubon D. Superiority of SDS lysis over saponin lysis for direct bacterial identification from positive blood culture bottle by MALDI-TOF MS. Proteomics Clin Appl. 2017;11(5–6). doi: 10.1002/prca.201600131 27921389

[35] Angeletti S, Dicuonzo G, D’Agostino A, Avola A, Crea F, Palazzo C, et al. Turnaround time of positive blood cultures after the introduction of matrix-assisted laser desorption-ionization time-of-flight mass spectrometry. New Microbiol. 2015;38(3):379–86. 26147149

[36] Maelegheer K, Nulens E. Same-day identification and antibiotic susceptibility testing on positive blood cultures: a simple and inexpensive procedure. Eur J Clin Microbiol Infect Dis. 2017;36(4):681–7. doi: 10.1007/s10096-016-2849-8 27889878

[37] Morgenthaler NG, Kostrzewa M. Rapid identification of pathogens in positive blood culture of patients with sepsis: review and meta-analysis of the performance of the sepsityper kit. Int J Microbiol. 2015;2015:827416. doi: 10.1155/2015/827416 26000017 PMC4426779

[38] Ferroni A, Suarez S, Beretti J-L, Dauphin B, Bille E, Meyer J, et al. Real-time identification of bacteria and Candida species in positive blood culture broths by matrix-assisted laser desorption ionization-time of flight mass spectrometry. J Clin Microbiol. 2010;48(5):1542–8. doi: 10.1128/JCM.02485-09 20237092 PMC2863924

[39] Loonen AJM, Jansz AR, Stalpers J, Wolffs PFG, van den Brule AJC. An evaluation of three processing methods and the effect of reduced culture times for faster direct identification of pathogens from BacT/ALERT blood cultures by MALDI-TOF MS. Eur J Clin Microbiol Infect Dis. 2012;31(7):1575–83. doi: 10.1007/s10096-011-1480-y 22080416 PMC3364411

[40] Martiny D, Dediste A, Vandenberg O. Comparison of an in-house method and the commercial Sepsityper™ kit for bacterial identification directly from positive blood culture broths by matrix-assisted laser desorption-ionisation time-of-flight mass spectrometry. Eur J Clin Microbiol Infect Dis. 2012;31(9):2269–81. doi: 10.1007/s10096-012-1566-1 22367290

[41] Buchan BW, Riebe KM, Ledeboer NA. Comparison of the MALDI Biotyper system using Sepsityper specimen processing to routine microbiological methods for identification of bacteria from positive blood culture bottles. J Clin Microbiol. 2012;50(2):346–52. doi: 10.1128/JCM.05021-11 22162549 PMC3264150

[42] Meex C, Neuville F, Descy J, Huynen P, Hayette M-P, De Mol P, et al. Direct identification of bacteria from BacT/ALERT anaerobic positive blood cultures by MALDI-TOF MS: MALDI Sepsityper kit versus an in-house saponin method for bacterial extraction. J Med Microbiol. 2012;61(Pt 11):1511–6. doi: 10.1099/jmm.0.044750-0 22837218

[43] Chen JHK, Ho P-L, Kwan GSW, She KKK, Siu GKH, Cheng VCC, et al. Direct bacterial identification in positive blood cultures by use of two commercial matrix-assisted laser desorption ionization-time of flight mass spectrometry systems. J Clin Microbiol. 2013;51(6):1733–9. doi: 10.1128/JCM.03259-12 23515548 PMC3716069

[44] Leli C, Cenci E, Cardaccia A, Moretti A, D’Alò F, Pagliochini R, et al. Rapid identification of bacterial and fungal pathogens from positive blood cultures by MALDI-TOF MS. Int J Med Microbiol. 2013;303(4):205–9. doi: 10.1016/j.ijmm.2013.03.002 23602511

[45] Kuo S-F, Huang T-Y, Lee C-Y, Lee C-H. Comparison of the Direct Identification and Short-Term Incubation Methods for Positive Blood Cultures via MALDI-TOF Mass Spectrometry. Diagnostics (Basel). 2024;14(15):1611. doi: 10.3390/diagnostics14151611 39125487 PMC11311331

[46] Ashmawy MM, Khairat SM, Reda NM, Abdelhalim MM. Rapid and cost-effective identification of microorganisms from positive blood cultures using MALDI-TOF MS. BMC Infect Dis. 2025;25(1):736. doi: 10.1186/s12879-025-11129-5 40410737 PMC12102807

[47] Matúšková LS, Vladárová M, Nováková E. Rapid Bacterial Identification and Quantitative Antimicrobial Susceptibility Assessment from Positive Blood Cultures to Optimize Bloodstream Infection Management. Microorganisms. 2026;14(3):633. doi: 10.3390/microorganisms14030633 41900392 PMC13028864

[48] Ferreira L, Sánchez-Juanes F, Porras-Guerra I, García-García MI, García-Sánchez JE, González-Buitrago JM, et al. Microorganisms direct identification from blood culture by matrix-assisted laser desorption/ionization time-of-flight mass spectrometry. Clin Microbiol Infect. 2011;17(4):546–51. doi: 10.1111/j.1469-0691.2010.03257.x 20456452

[49] Jamal W, Saleem R, Rotimi VO. Rapid identification of pathogens directly from blood culture bottles by Bruker matrix-assisted laser desorption laser ionization-time of flight mass spectrometry versus routine methods. Diagn Microbiol Infect Dis. 2013;76(4):404–8. doi: 10.1016/j.diagmicrobio.2013.04.013 23726652

[50] Nonnemann B, Tvede M, Bjarnsholt T. Identification of pathogenic microorganisms directly from positive blood vials by matrix-assisted laser desorption/ionization time of flight mass spectrometry. APMIS. 2013;121(9):871–7. doi: 10.1111/apm.12050 23331371

[51] Spanu T, Posteraro B, Fiori B, D’Inzeo T, Campoli S, Ruggeri A, et al. Direct maldi-tof mass spectrometry assay of blood culture broths for rapid identification of Candida species causing bloodstream infections: an observational study in two large microbiology laboratories. J Clin Microbiol. 2012;50(1):176–9. doi: 10.1128/JCM.05742-11 22090401 PMC3256727

[52] Yan Y, He Y, Maier T, Quinn C, Shi G, Li H, et al. Improved identification of yeast species directly from positive blood culture media by combining Sepsityper specimen processing and Microflex analysis with the matrix-assisted laser desorption ionization Biotyper system. J Clin Microbiol. 2011;49(7):2528–32. doi: 10.1128/JCM.00339-11 21543564 PMC3147835

[53] Drancourt M. Detection of microorganisms in blood specimens using matrix-assisted laser desorption ionization time-of-flight mass spectrometry: a review. Clin Microbiol Infect. 2010;16(11):1620–5. doi: 10.1111/j.1469-0691.2010.03290.x 20545958

[54] Lavergne R-A, Chauvin P, Valentin A, Fillaux J, Roques-Malecaze C, Arnaud S, et al. An extraction method of positive blood cultures for direct identification of Candida species by Vitek MS matrix-assisted laser desorption ionization time of flight mass spectrometry. Med Mycol. 2013;51(6):652–6. doi: 10.3109/13693786.2012.762607 23373445

[55] Marinach-Patrice C, Fekkar A, Atanasova R, Gomes J, Djamdjian L, Brossas J-Y, et al. Rapid species diagnosis for invasive candidiasis using mass spectrometry. PLoS One. 2010;5(1):e8862. doi: 10.1371/journal.pone.0008862 20111603 PMC2810333

[56] Campigotto A, Goneau L, Matukas LM. Direct identification and antimicrobial susceptibility testing of microorganisms from positive blood cultures following isolation by lysis-centrifugation. Diagn Microbiol Infect Dis. 2018;92(3):189–93. doi: 10.1016/j.diagmicrobio.2018.06.010 30122510

[57] Kim J-H, Kim TS, Song SH, Choi J, Han S, Kim DY, et al. Direct rapid antibiotic susceptibility test (dRAST) for blood culture and its potential usefulness in clinical practice. J Med Microbiol. 2018;67(3):325–31. doi: 10.1099/jmm.0.000678 29458541

[58] Pulido MR, García-Quintanilla M, Martín-Peña R, Cisneros JM, McConnell MJ. Progress on the development of rapid methods for antimicrobial susceptibility testing. J Antimicrob Chemother. 2013;68(12):2710–7. doi: 10.1093/jac/dkt253 23818283

[59] de Cueto M, Ceballos E, Martinez-Martinez L, Perea EJ, Pascual A. Use of positive blood cultures for direct identification and susceptibility testing with the vitek 2 system. J Clin Microbiol. 2004;42(8):3734–8. doi: 10.1128/JCM.42.8.3734-3738.2004 15297523 PMC497633

[60] Quesada MD, Giménez M, Molinos S, Fernández G, Sánchez MD, Rivelo R, et al. Performance of VITEK-2 Compact and overnight MicroScan panels for direct identification and susceptibility testing of Gram-negative bacilli from positive FAN BacT/ALERT blood culture bottles. Clin Microbiol Infect. 2010;16(2):137–40. doi: 10.1111/j.1469-0691.2009.02907.x 19778301

[61] Liang B, Li X, Zheng Q, Cai H, Zhu S, Zhang M, et al. Evaluation of three rapid antimicrobial susceptibility testing methods directly from positive blood cultures integrated with rapid pathogen identification. BMC Microbiol. 2026;26(1):237. doi: 10.1186/s12866-025-04697-8 41629772 PMC12980964

